# Effect of melatonin on bovine sperm characteristics and ultrastructure changes following cryopreservation

**DOI:** 10.1002/vms3.224

**Published:** 2019-12-02

**Authors:** A. R. ChaithraShree, Shailesh D. Ingole, Vikas D. Dighe, Anagha S. Nagvekar, Simin V. Bharucha, Nilesh R. Dagli, Prakash M. Kekan, Shambhudeo D. Kharde

**Affiliations:** ^1^ Department of Animal Husbandry Hassan Karnataka India; ^2^ Department of Veterinary Physiology Bombay Veterinary College Parel Mumbai India; ^3^ National Center for Preclinical Reproductive and Genetic Toxicology NIRRH Mumbai India; ^4^ Teaching Veterinary Clinical Complex Bombay Veterinary College Parel Mumbai India

**Keywords:** bulls, Cryopreservation, Melatonin, Ultrastructure changes

## Abstract

The production of reactive oxygen species (ROS) during cryopreservation of semen alters the sperm motion and mobility characteristics, resulting in poor or failure of conception rate after artificial insemination (AI). Melatonin an antioxidant is able to modulate the effect of ROS and prevents spermatozoa by reducing the oxidative stress during freezing process. Eight ejaculates from eight healthy HF bulls diluted with Tris egg yolk glycerol extender were divided into five equal aliquots. The Computer Assisted Semen Analyser (CASA) results showed no significant difference between the control—post‐ thaw samples and melatonin‐treated samples; however, the velocity of spermatozoa with regard to the VAP, VSL showed highest increase in the 0.25 mM MLT‐treated semen followed by 0.1 mM MLT treated semen except for VCL where velocity increased with increase in the concentration of melatonin. The vigour of spermatozoa regard to BCF, STR and LIN recorded highest increase in the 0.25 mM MLT treated semen followed by 0.1 mM MLT‐treated semen except for the ALH where vigour increased with increase in the concentration of melatonin. The electron micrography images illustrated that the addition of 0.1 mM melatonin protected the plasma membrane and acrosome region and maintained the ultrastructure integrity of the cryopreserved spermatozoa when compared to control group, whereas the electron micrography of spermatozoa treated with 0.2 and 0.25 mM melatonin illustrated highest damage to the plasma and acrosome membrane. Thus concluding that inclusion of melatonin to sperm extender can improve the post‐thaw quality of cryopreserved bull spermatozoa.

## Graphical abstract

The production of ROS during cryopreservation of semen alters the sperm motion and mobility characteristics, resulting in poor or failure of conception rate after AI. Melatonin an antioxidant is able to modulate the effect of ROS and prevents spermatozoa by reducing the oxidative stress during freezing process. The electron micrography images illustrated that the addition of 0.1 mM melatonin protected the plasma membrane and acrosome region and maintained the ultrastructure integrity of the cryopreserved spermatozoa indicating inclusion of melatonin to sperm extender can improve the post‐thaw quality of cryopreserved bull spermatozoa

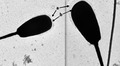

## 1 INTRODUCTION

AI is the predominant methodology used in bovine reproduction around the world. Main objective of the dairy farm is to produce one calf per cow annually. An important element of economical calf production is top quality bull semen for successful fertilization and improved herd health (Swanson & Herman, 1940). Generation of reactive oxygen species (ROS) throughout freeze thaw cycle accompanied by low antioxidant levels in seminal plasma and in extender induces a state of oxidative stress that cause lipid peroxidation of the bio‐membrane system and result in reduced semen quality. High levels of ROS could decline spermatozoan motility, inactivate glycolytic enzymes have an effect on semen quality by deteriorating membrane lipids, proteins and nuclear/mitochondrial DNA (Asma‐ul‐Husna et al., 2017). Although bovine semen has natural defence system against the ROS, it is considered insufficient under cryopreservation‐mediated stress (Nichi et al., 2006). Supplementation of extender with appropriate antioxidant is recommended to reduce oxidative damage during freeze‐thawing of bull spermatozoa.

Melatonin an indoleamine neurohormone synthesized mainly in the pineal gland (Boutin, Audinot, Ferry, & Delagrange, 2005) is an intracellular antioxidant that protects the cells from ROS‐mediated damages under oxidative stress each in vivo and in vitro (Asma‐ul‐Husna et al., 2017). Recently, supplementation of melatonin as a potent antioxidant has been implicit either througout cryopreservation of semen (Zhao et al., 2015). More specifically, melatonin can directly benefit sperm characteristics, together with increased total motility, progressive motility, and viability rate enhanced sperm membrane and DNA integrity reduced membrane lipid peroxidation and modulation of sperm capacitation (Pang et al., 2016). The effect of melatonin on bovine spermatozoa remains arguable (Ashrafi, Kohram, & Ardabili, 2013) and therefore underlying mechanism has not nevertheless been well elucidated. However, the effect of melatonin on HF cross bull spermatozoa was seldom evaluated. Therefore, this study aimed to estimate the influence of melatonin on the sperm motility characteristics and fine ultrastructure changes of the HF cross bull spermatozoa after 48 hr of cryopreservation.

## 2 MATERIALS AND METHODS

Prior approval of the Institutional Animal Ethics Committee was obtained for the experimental procedures.

### 2.1 Animals, sample collection and processing of ejaculates

Semen samples obtained from eight healthy fertile HF cross bulls (aged 4–8 years) maintained at Frozen Semen Center, DCKL, Mumbai, were assessed for volume, mass activity, sperm concentration and percentage of motile spermatozoa. The ejaculates with atleast 70% motility, 820 × 10^6^ sperm cells/ml and >85% normal sperm morphology were used for the present study. After evaluation of semen quality, the fresh semen samples were diluted with Tris Egg Yolk Glycerol extender. Each extended ejaculate was divided into 5 equal portions and supplemented with different concentrations of melatonin (Melatonin Extrapure, 5 gms, SRL, India). Group 1: semen without melatonin (control—pre‐freeze), group 2: semen without melatonin (control—post‐thaw), group 3 to group 5 semen with 0.1 mM, 0.2 mM and 0.25 mM of melatonin, respectively. Melatonin stock solution was prepared by diluting the melatonin powder in deionized water.

The extended semen samples were filled in 0.5 ml French medium polyvinyl chloride straws and sealed under laminar flow unit. The group 1 semen samples (without melatonin) were cooled slowly to 5°C and were carried to laboratory for pre‐freeze assessment of transmission electron micrography. Group 2 to group 5 semen straws were frozen in liquid nitrogen (−196°C) for cryopreservation. These straws were stored for 48 hr before the evaluation and then thawed and analysed for sperm motility parameters using CASA and ultrastructure changes by TEM.

#### 2.1.1 Freezing of straws

The equilibrated straws were placed on the racks which were placed on grill present in L.R. 320 (union carbide) container. Level of liquid nitrogen (LN_2_) was kept upto grill, 20 min before freezing. The straws and gelatin containers were frozen 4 cm above LN_2_ level for 10 min to achieve temperature of −140°C. Thus, the average freezing rate was −14.5°C per minute. The straws and gelatin containers on each rack were collected and quickly immersed in respective goblets containing liquid nitrogen and these goblets were transferred to smaller LN_2_ storage container.

#### 2.1.2 Thawing of Semen

The French medium straws were removed from liquid nitrogen container after 48 hr of cryopreservation and thawed in water bath at 37˚C for 30 s.

### 2.2 Assessment of sperm motilities by computer assisted semen analyser (CASA)

A CASA (Version 12.3, IVOS, Hamilton Thorne Research) was used to analyse total motile spermatozoa (%TM), the velocity average pathway (VAP:µm/s), the velocity straight line (VSL:µm/s), the velocity of curved line (VCL:µm/s), the amplitude lateral head (ALH:µm), the beta cross frequency (BCF:Hz), the straightness (STR:%) and the linearity (LIN:%). The software settings were arranged as follows. Chamber type: Leja 4 (20 µM), temperature of analysis (˚C): 37.0, fields acquired: 1, video source: 60 Hz (dark field), illumination intensity: 2,151 and magnification: 0.75. Immediately after gentle mixing of semen samples (pre‐freeze/ post‐thaw), 10 μl of sample was pipetted and loaded into a pre‐warmed (37°C) dual chamber Leja slide and covered with a cover slip and was left for some time to settle on the mini‐therm heating stage before the analysis. Sperm motility was assessed with a microscope equipped with a 10X negative‐phase contrast objective and analysis was based on the examination of consecutive digitalized images obtained from several fields.

### 2.3 Hypo osmotic swelling test (HOST)

0.1 ml of semen was added to 1 ml of media (Sodium citrate dehydrate—7.35g, Fructose—13.51g, Distilled water—100 ml) and incubated at 37°C for 30 min. A drop of this mixture was placed on the clean glass slide and was observed under 100X and 200 sperms were counted in at least five different fields and the percentage of swollen sperms was counted.

### 2.4 Assessment of acrosome membrane intactness

A thin smear of semen (pre‐freeze/post‐thaw) was prepared, air dried and kept in 5% formaldehyde for 30 min at 37°C temperature. The slide was washed in distilled water and air dried. The air dried samples were kept in 3 ml staining solution (Giemsa stain—1g, Methanol—66 ml, Glycerol—60 ml) mixed with 2 ml of buffer at pH 6.8 and 45 ml double distilled water for 3 hr at 37°C. The stained slides were washed with distilled water and air dried and examined under 100X.

### 2.5 Ultrastructure analysis of spermatozoa

The ultrastructure changes in the spermatozoa were evaluated by TEM. After centrifugation of semen samples at 978 *g* for 15 min, the supernatant was discarded and sediment was fixed with 1ml of Karnovsky's fixative (4% Gluteraldehyde + 4% paraformaldehyde + 0.02% picric acid + 0.02% calcium chloride + 0.2% Mcacodylate buffer). The fixed bull semen sample was suspended in 1ml 0.1M sodium cacodylate buffer. The sample was further diluted to 1:2 times with distilled water. Different solutions of the samples were taken on formvar carbon‐coated copper grids and incubated for 10min, there after strained with 2% uranyl acetate for 10min. The sample grids were washed with distilled water, dried and visualized under TEM (TECNAI 12BT, 120 KV, FEI) and photographed for further analysis.

### 2.6 Statistical analysis

Data were statistically analysed using Complete Randomized Design (CRD) according to Snedecor and Cochran, (1998). Differences in means were tested using CD test in order to study the effect of melatonin on sperm motility parameters in HF cross bulls.

## 3 RESULTS

### 3.1 Sperm motility assessment by CASA

The addition of melatonin did not have any effect on the motility parameters of frozen‐thawed bull semen, as there have been no statistically significant differences determined between the treatment and control groups (Table 1).

**Table 1:** Sperm motility parameters of cryopreserved bull spermatozoa without and with addition of melatonin

Concentration of melatonin	TM (%)	VAP (µm/s)	VSL (µm/s)	VCL (µm/s)	ALH (µm)	BCF (Hz)	STR (%)	LIN (%)
Control pre‐freeze	72.62 ± 8.18	251.43 ± 32.94	202.01 ± 24.43	407.30^a^ ± 57.73	14.75^a^ ± 2.11	12.65 ± 2.47	80.50^c^ ± 1.61	53.12^c^ ± 3.14
Control post‐thaw	62.00 ± 10.16	158.47 ± 37.15	150.57 ± 36.67	197.21^b^ ± 33.59	5.48^b^ ± 1.23	14.46 ± 3.54	93.00^a^ ± 2.19	75.25^a^ ± 7.47
0.1 mM	74.00 ± 8.25	167.02 ± 31.70	150.58 ± 32.05	215.13^b^ ± 35.15	7.07^b^ ± 1.50	14.46 ± 3.54	90.50^ab^ ± 2.22	71.85^ab^ ± 5.68
0.2 mM	50.25 ± 7.79	156.41 ± 25.45	128.67 ± 22.62	235.51^b^ ± 37.70	9.28^b^ ± 2.53	15.92 ± 3.20	85.12^bc^ ± 3.90	58.62^bc^ ± 5.61
0.25 mM	84.00 ± 7.47	245.55 ± 34.70	233.63 ± 35.02	283.43^b^ ± 33.76	6.21^b^ ± 0.89	21.83 ± 3.34	94.62^a^ ± 2.22	82.62^a^ ± 5.44

Note

Mean with at least one common superscript do not differ significantly at 5% level. Mean ± standard error shown.

Abbreviations: ALH, amplitude of lateral head displacement; BCF, beta cross frequency; LIN, linearity; STR, straightness; TM, percentage of total motile sperm; VAP, average path velocity; VCL, curvilinear velocity; VSL, straight line velocity.

The TM (%) of spermatozoa disclosed that there was no important distinction between control and melatonin treated semen samples. It decreased numerically in control group after cryopreservation than in before cryopreservation. However, the motility of spermatozoa treated with different concentrations of melatonin accrued numerically (except for 0.2 mM) with respect to control group after cryopreservation. The highest motility percentage was seen in 0.25 mM melatonin (84.00 ± 7.47%) treated semen followed by 0.1 mM melatonin (74.00 ± 8.25%) treated samples.

The VAP of the spermatozoa was lowered non‐significantly in control—post‐thaw as compared to control—pre‐freeze group. However, within the melatonin‐treated groups, the mean VAP (µm/sec) value increased (except for 0.2 mM) non‐significantly with respect to control group after cryopreservation. The highest VAP was within the samples treated with 0.25 mM melatonin (245.55 ± 34.70) followed by samples treated with 0.1 mM melatonin (167.02 ± 31.70).

The VSL indicated no significant difference between groups, however VSL decreased numerically in control—post‐thaw as compared to control—pre‐freeze (150.57 ± 36.67 and 202.01 ± 24.43 µm/s, respectively). After cryopreservation, the mean VSL nearly remained similar in control—post‐thaw and 0.1 mM melatonin (150.58 ± 32.05 µm/s) treated group, however, decreased in 0.2 mM melatonin (128.67 ± 22.62 µm/s) treated group. The highest VSL was observed in 0.25 mM (233.63 ± 35.02 µm/s) melatonin‐treated group.

The VCL revealed a significant decrease (*p* < .05) among the melatonin treated and untreated (control—post‐thaw) groups (197.21 ± 33.59) in comparison with diluted control—pre‐freeze (407.30 ± 57.73). However, the VCL in melatonin‐treated groups increased non‐significantly with respect to control—post‐thaw group after cryopreservation. The highest VCL (µm/s) was maintained with 0.25 mM (283.43 ± 33.76) melatonin followed by 0.2 and 0.1 mM melatonin.

The ALH significantly decreased (*p* < .05) in all melatonin treated and untreated (control—post‐thaw) groups in comparison with control—pre‐freeze group. It was observed that, within the melatonin treated groups, the ALH increased non‐significantly with respect to control group after cryopreservation. The highest ALH was observed in 0.2 mM melatonin followed by 0.1 mM melatonin‐treated group.

There was no significant difference within the mean BCF values of the spermatozoa treated with melatonin and control—post‐thaw group in comparison with control—pre‐freeze group. However, the values non‐significantly increased in all melatonin treated and untreated (control—post‐thaw) groups after cryopreservation. The highest BCF was observed in 0.25 mM melatonin followed by 0.1 mM melatonin with respect to control group.

The percentage of straightness increased significantly (*p* < .05) in all melatonin treated and untreated (post‐thaw) groups in comparison with control—pre‐freeze group. However, the highest non‐significant STR was observed in 0.25 mM (94.62 ± 2.22%) melatonin with respect to control group after cryopreservation whereas the STR was significantly lower in 0.2 mM melatonin than the other melatonin groups.

Similar to STR, there was significant (*p* < .05) increase in the mean LIN (%) value of spermatozoa with different concentrations of melatonin‐treated group (except for 0.2 mM melatonin) and untreated (post‐thaw) group in comparison with control—pre‐freeze group. The highest LIN (%)was observed in 0.25 mM melatonin with respect to control group after cryopreservation.

### 3.2 Hypo osmotic swelling test (HOST)

**Figure 1:**
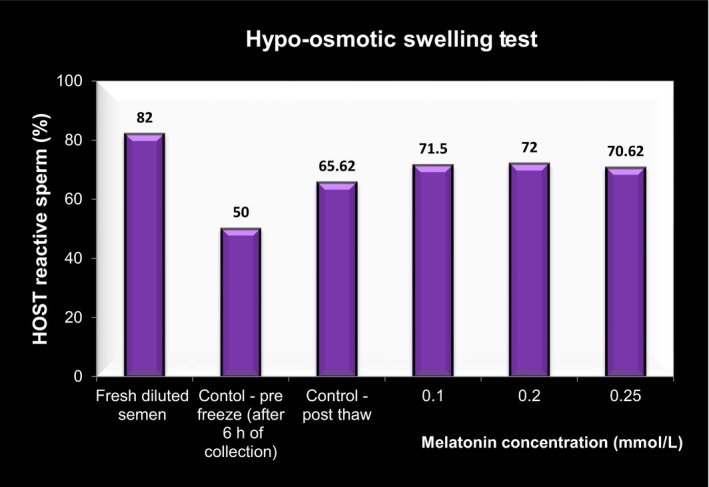
HOST reacted spermatozoa (%) with and without melatonin

**Figure 2:**
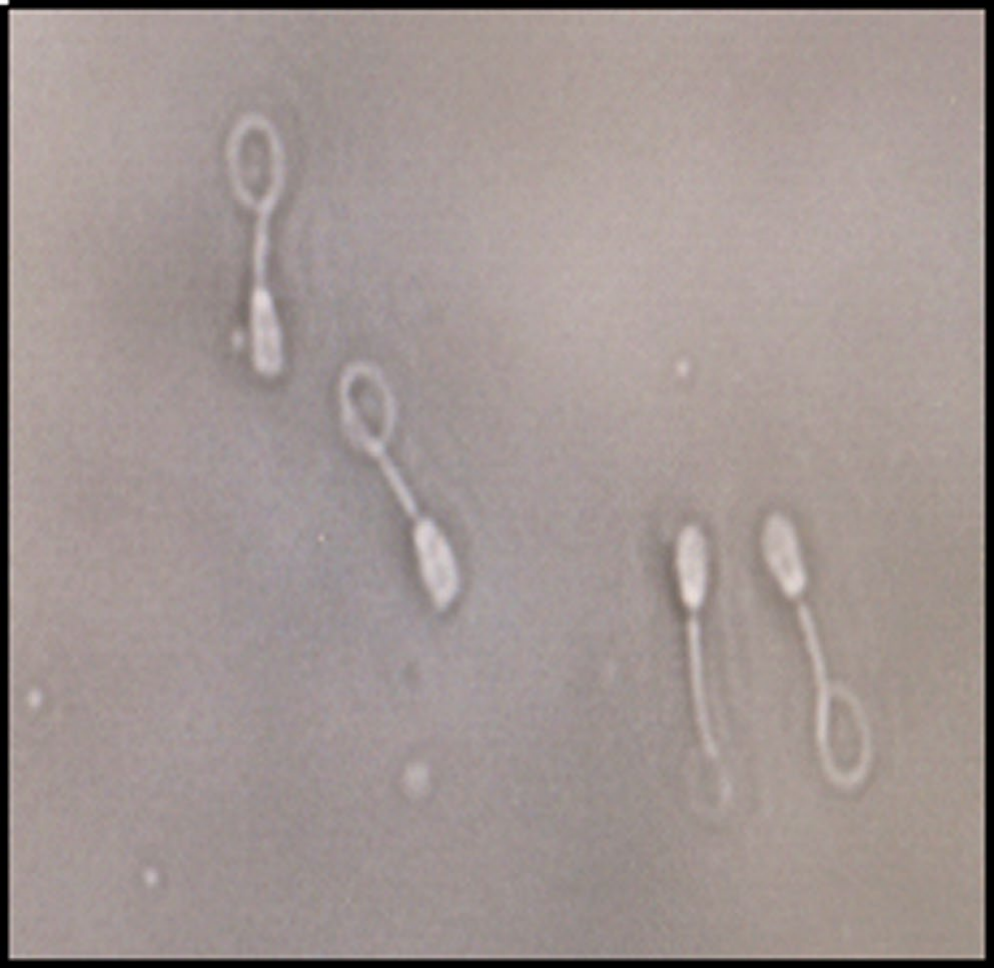
HOST reacted spermatozoa (coiled tail ‐ 100X)

**Figure 3:**
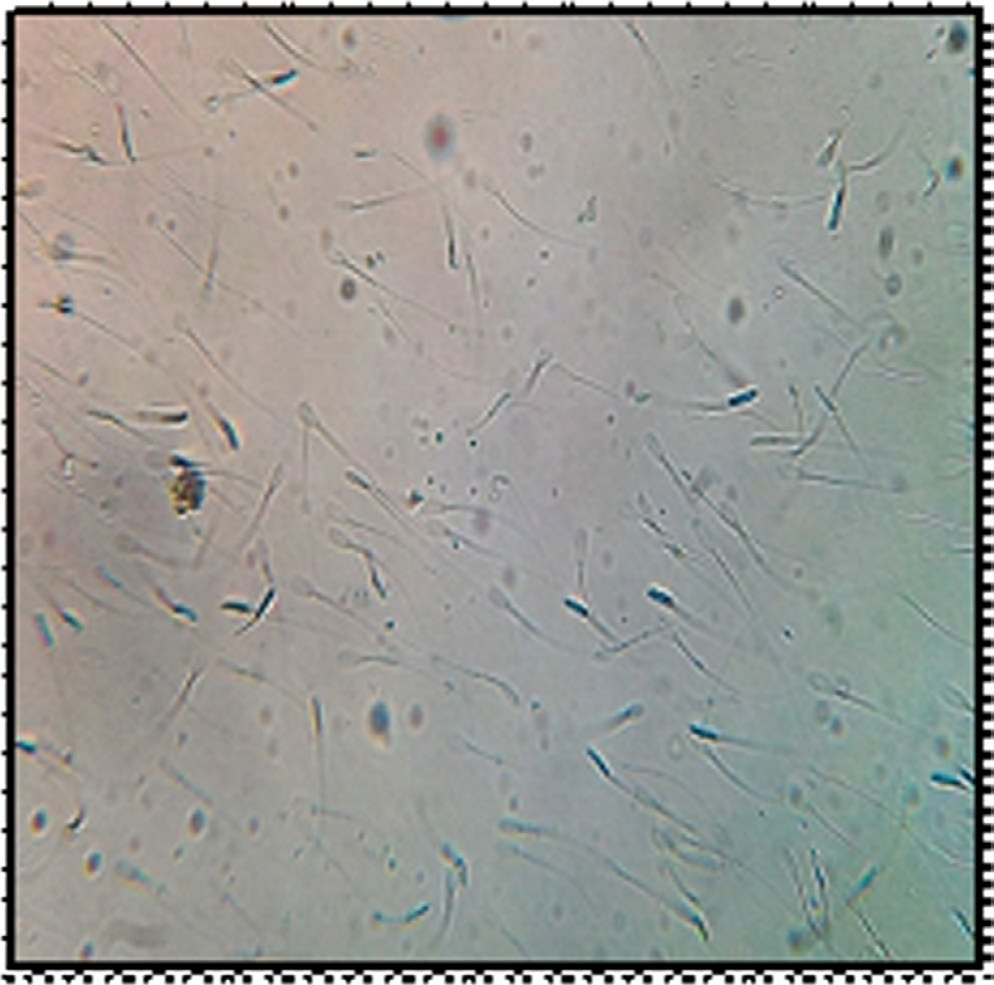
HOST non‐reacted spermatozoa (uncoiled tail ‐ 100X)

The percent HOST reactive spermatozoa are illustrated and depicted in Figures 1, 2 and 3. It is inferred that the percentage of HOST reactive spermatozoa increased significantly (*p* < .05) in all the melatonin treated and untreated samples (control—post‐thaw) when compared with control—pre‐freeze samples. The highest HOST reactive spermatozoa percent was observed in 0.2 mM MLT followed by 0.1 mM MLT with respect to control group after cryopreservation. The lower percentage in control pre‐freeze without melatonin was due to the time taken for transferring of the semen sample from the collection centre to the laboratory.

### 3.3 Acrosome membrane integrity/ acrosome intactness

**Figure 4:**
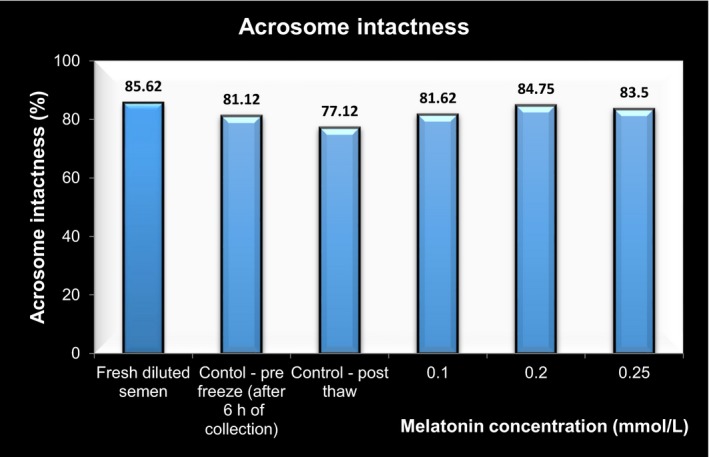
Percentage of acrosome intactness with and without melatonin

**Figure 5:**
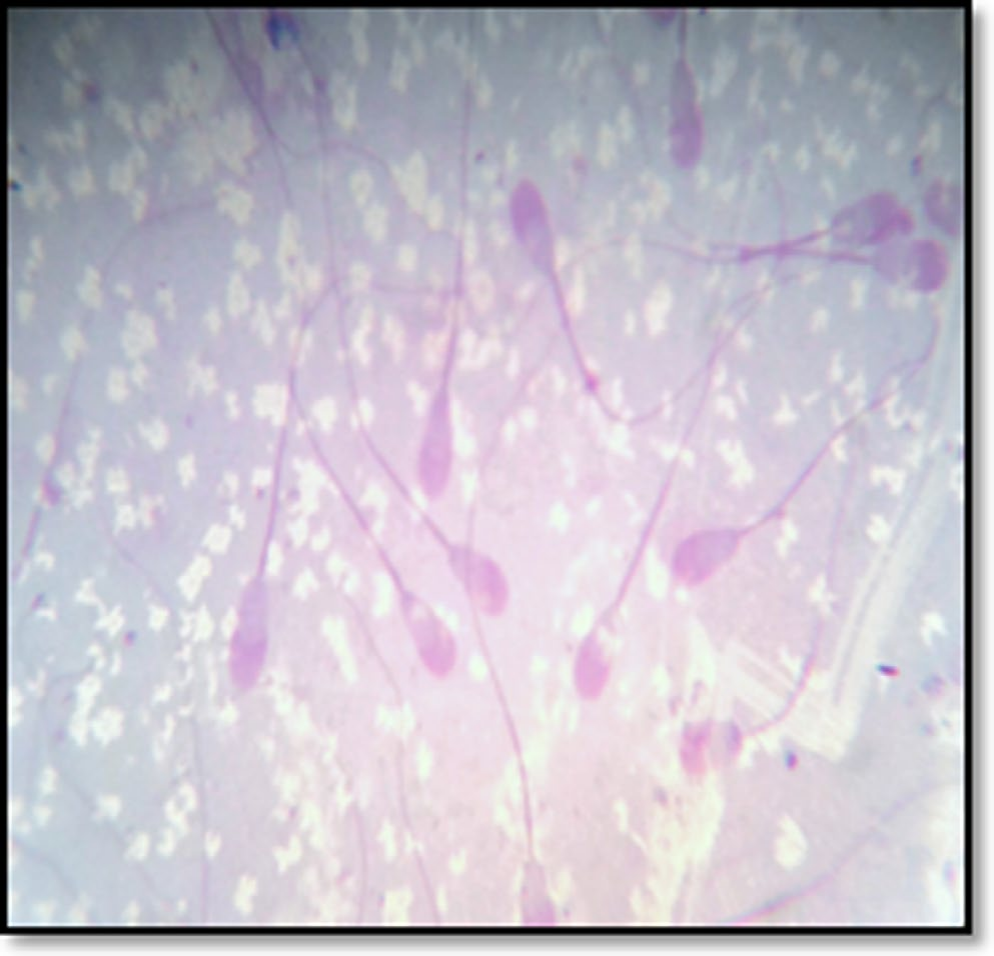
Spermatozoa with intact acrosome (reacted) (Giemsa stain – 100X)

The acrosome intactness of spermatozoa is illustrated and depicted in Figures 4 and 5. It revealed that there was significant increase (*p* < .05) in the mean intact acrosome percentage in the entire melatonin treated group in comparison with control—post‐thaw group. The highest percentage of spermatozoa with intact acrosome was observed in 0.2 mM MLT followed by 0.25 mM MLT with respect to control group after cryopreservation.

### 3.4 Ultrastructure changes of cryopreserved spermatozoa

**Figure 6:**
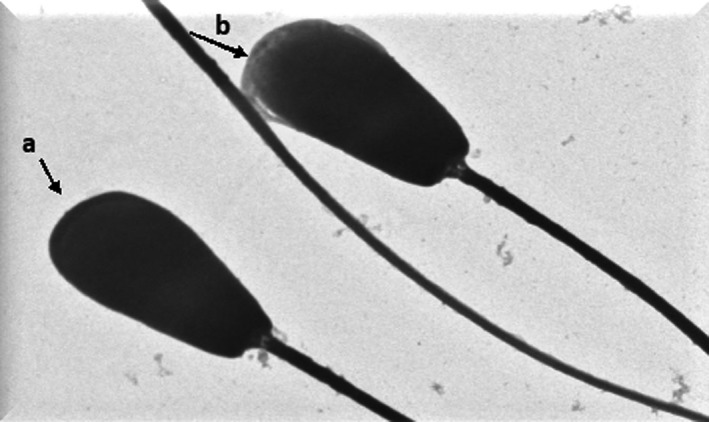
Electron micrograph of control post‐thaw (without melatonin) sperm cells (a) with no plasma membrane and (b) with altered acrosome membrane

**Figure 7:**
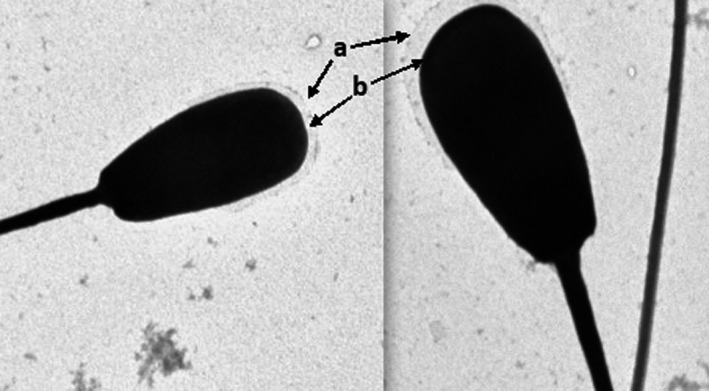
Electron micrograph of sperm cells treated with 0.1 mM melatonin illustrating (a) intact plasma membrane and (b) acrosome membrane

**Figure 8:**
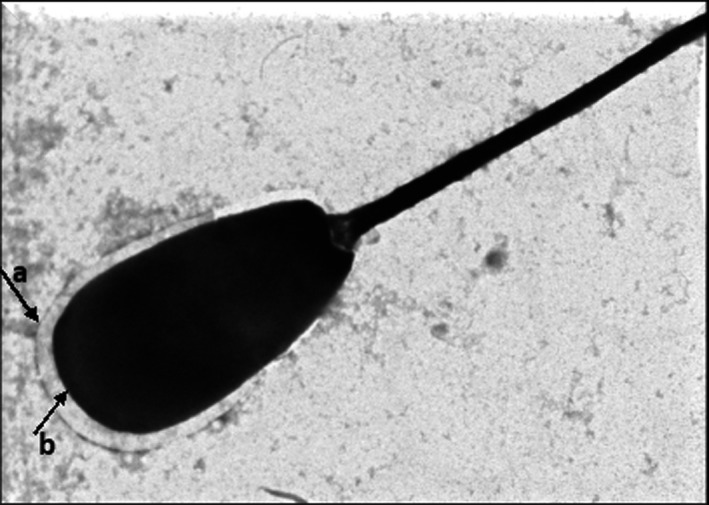
Electron micrograph of pre‐freeze (control) sperm cell (a) with intact plasma membrane and (b) with acrosome membrane

**Figure 9:**
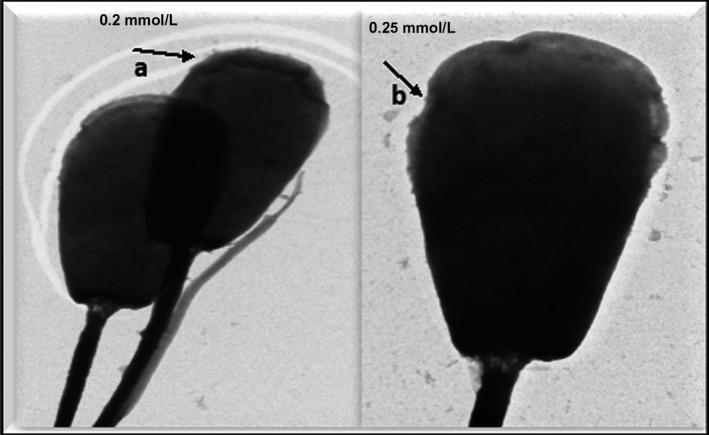
Electron micrograph of sperm cells treated with 0.2 mM and 0.25 mM melatonin showing (a) absence of plasma membrane and (b) highly altered acrosome membranes

The electron microscopic images of the HF cross bull sperm cells in the post‐thaw control group (Figure 6) showed absence of plasma membrane and altered acrosome membranes. The thawed spermatozoa treated with 0.1 mM melatonin (Figure 7) illustrated well defined and intact plasma membrane and acrosome membrane similar to those sperm cells in the pre‐freeze control group (Figure 8) with fine integrity of plasma and acrosome membranes. The semen samples treated with 0.25 mM melatonin revealed higher rate of damaged/absent plasma membrane and acrosome membranes followed by samples treated with 0.2 mM melatonin (Figure 9) indicating that the higher concentration of melatonin failed to maintain the fine structure of spermatozoa.

## 4 DISCUSSION

The antioxidant system of seminal plasma and spermatozoa is compromised during semen cryopreservation. Thus melatonin reduces the oxidative stress seen during freeze thawing process leading to increase in total motility percentage (Ashrafi et al., 2013).

However, the addition of melatonin tends to maintain sperm motility by stabilizing the integrity of acrosome and spermatozoa plasmalemma (Perumal, Chamuah, Nahak, & Rajkhowa, 2015). Melatonin in sperm cells scavenge several ROS directly for protecting mammalian cells against oxidative stress and thus maintain sperm motility (Karimfar et al., 2015). The additive might have displayed cryoprotective effect on the functional integrity of mitochondria that is responsible for the generation of energy from intracellular stores of ATP and thus improved post‐thaw sperm motility (Reddy, Jagan, & Atreja, 2010).

The data obtained regarding sperm motility are in accordance with Ashrafi et al., (2013), Cheuqueman et al., (2014), Abdel‐Khalek, El‐Nagar, and Omima (2016), Kapadiya, Nakhashi, Sutaria, Rathod, and Suthar (2016), Pang et al., (2016), Asma‐ul‐Husna et al., (2017) in bulls; Perumal, Vupru, and Khate (2013), Perumal et al., (2015), Perumal, Chang, Sangma, Savino, and Khate (2016a), Perumal, Baruah, et al. (2016b) in Mithun at 5°C; Ashrafi, Kohram, Naijian, Bahreini, and Poorhamdollah (2011), Succu et al., (2011), Casao et al., (2010) in rams; Balao da Silva et al., (2011) in stallions at 37°C; Izadpanah, Zare‐Shahneh, Zhandi, Yousefian, and Emamverdi (2015) in stallions at 5°C and El‐Battawy and El‐Nattat (2013) in goats; who reported that the addition of melatonin to semen freezing extender could protect the spermatozoa from damage during cryopreservation and improve the post‐thaw semen quality.

Melatonin has improved the velocity parameters in dose‐dependent manner though non‐significant. The improvement in the velocity parameters is due to interaction of melatonin with calmodulin which successively influences cytoskeletal elements of sperm and impacted higher sperm motility and velocity. Thus melatonin protects the sperm from stress and damage during the processing of dilution, extension and preservation. Melatonin in the sperm extender induces rapid velocity and elevates intracellular ATP concentration which is the main source of energy used by the sperm flagellum to initiate and activate forward progressive motility and different velocity parameters (Perumal, Chang, et al., 2016a).

The inclusion of melatonin at the rate of 0.1 mM and 0.25 mM has improved the mobility and velocity parameters of HF cross bull semen during cryopreservation. This may be due to the tendency of melatonin to stimulate the cellular influx of Ca^2+^ into sperm cells enhancing their motility (Delgadillo, Tay, & King, 1994). Calcium and its channels control the transition from symmetric to asymmetric flagella wave forms (Si, 1997) and calmodulin acts as an intracellular regulator of Ca^2+^ function and have been identified both in the head and flagellar parts of spermatozoa (Tash & Means, 1983). Calmodulin antagonist or lack of melatonin causes a reduction in VCL and it is related with mitochondrial membrane potential and mobility and velocity of the spermatozoa (Ahmad, Bracho, Wolf, & Tash, 1996). Moreover melatonin has improved the velocity through act on the cAMP stimulator (Yung, Sim, & Wong, 1995) and cAMP stimulates sperm motility via its direct action on the axoneme of the tail (Lindemann, 1978) or indirectly through acting on the cell membrane as secondary messenger (Garbers & Kopf, 1908).

Melatonin increased the sperm velocity attributes because it decreased significantly the sperm abnormalities and acid phosphatase level during preservation in the extender since it can pass into the cell membrane and protects mitochondria and DNA from free radical and ROS damaging effect through its potent antioxidant and anti‐ageing effects on the cells (Perumal, Chang, et al., 2016a). Thus melatonin improves sperm progressive motility as it has decreasing effect on the phosphatase enzyme release and leakage from sperm cells during preservation. The increase in velocity parameters for melatonin treated spermatozoa could be due to its effect through specific receptors. Therefore, addition of melatonin improves velocity parameters, possibly by reducing LPO and increasing total antioxidant capacity and antioxidant enzyme activity (Pang et al., 2016).

The HOST results in the present study are in conformation with the studies of Ashrafi et al. (2013), El‐Raey, Badr, Assi, & Rawash (2015) in bulls; Perumal et al. (2013), Perumal et al. (2015) in Mithun; Chankitisakul (2014) in boars at 15˚C and Izadpanah et al. (2015) in stallions; in which plasma membrane integrity improved with the addition of melatonin.

The inclusion of melatonin increased sperm plasma membrane integrity following the freeze–thawing process. The chemical and physical effects of these reagents/processes may cause extensive cryodamage to plasma membranes with resultant changes in their normal functions (Keel & Webster, 1993). The increase in HOST reactive sperm may be due to melatonin‐induced maintenance of plasma and mitochondrial membrane integrity and cytoskeletal structure of flagella of sperm by stimulating the activities of antioxidant enzymes (Perumal et al., 2015). The melatonin supplementation helps in eliminating the toxic ROS (Asma‐ul‐Husna et al., 2017). Thus melatonin by facilitating the activities of several enzymes involved in metabolizing ROS, preserves cell membrane fluidity (Garcia et al., 1997; Okatani, Wakatsuki, & Kaneda, 2000; Reiter et al., 2009) as well as protects the mitochondria (Balao da Silva et al., 2011).

The results of acrosomal integrity in melatonin treated semen are similar to those reported by Cheuqueman et al. (2014), Kapadiya et al. (2016), Pang et al. (2016) in bulls; Perumal et al. (2013), Perumal et al. (2015) in Mithun; El‐Raey, Badr, Rawash, and Darwish (2014) in goats and Martin‐Hidalgo et al. (2011) in boars. The present study is in conclusion of melatonin capacity in shielding acrosomal characters by keeping check on hazardous effects encountered during cryopreservation.

Oxidative stress control has a physiological impact on reproductive processes; low free radical levels modulate gamete function, particularly capacitation and chemotactic acquisition by spermatozoa. Cryopreservation causes permanent functional damage to sperm viability that can be explained partially by the reduction in the percentage of normal intact acrosomes and in total acrosin activity (Asma‐ul‐Husna et al., 2017).

The protective role exerted by melatonin is to its antioxidant effects. The small size of melatonin allows it to pass through morpho‐physiological barriers with ease and to distribute uniformly across various cellular components, including the cytosol, mitochondria and nucleus. The presence of melatonin receptors, MT1 and MT2 in sperm implies that melatonin supplementation can directly influence sperm function, perhaps by up‐regulating antioxidant defences. Melatonin administration improves post‐thawed intact acrosome rates that could attributed to its decreasing effect on the phosphatase enzyme release and leakage from sperm cells during cryopreservation (El‐Battawy et al., 2013). Enhanced acrosomal integrity due to addition of MLT on spermatozoa might be due to a potent scavenging effect of MLT on NO and ROS that directly and indirectly affect the sperm viability by triggering the sperm apoptosis (El‐Raey et al., 2014) and stabilization of plasmalemma of spermatozoa, thus increasing the motility (Perumal et al., 2015).

The electron micrography revealed that the addition of 0.1 mM melatonin maintained the fine structure of plasma membrane and acrosome integrity. However, 0.25 mM melatonin caused higher damage. Various effect of melatonin on the integrity of plasma and acrosome membrane at various doses in the present study may be due to the excessive amount of melatonin than optimum in turn to higher fluidity of plasma membrane of sperm, creating the sperm more prone to plasma membrane and acrosomal damages and also inclusion of high dosage leads to deleterious effect on the spermatozoa as because alteration in physiological and physical condition of diluent (Perumal, Chang, et al., 2016a). The plasma membrane of mammalian spermatozoa contains high concentrations of arachidonic and decosahexaenoic acids, that create it prone to ROS iatrogenic peroxidative injury with a later loss of sperm functions (Lenzi et al., 2002; Nair, Brar, Ahuja, Sangha, & Chaudhary, 2006). Destruction of integrity causes rise in the membrane permeability and decrease in the ability of sperm to control the intracellular concentration of ions that in turn are involved in sperm motion (Baumber, Ball, Gravance, Medina, & Davies‐Morel, 2000).

Present finding regarding ultrastructure changes of sperm cells of HF bulls are in close agreement with those recorded in Egyptian buffalo bulls (El‐Raey et al., 2015) indicating the role of melatonin in preserving the integrity of plasma membrane and acrosome membrane (0.1 mM concentration) of frozen spermatozoa. Subtle anomalies in the ultra‐structures were only assessed by TEM. Melatonin is known to stimulate the activities of several enzymes involved in metabolizing ROS and thereby preserves cell membrane fluidity (Garcia et al., 1997; Okatani et al., 2000). While free radicals damage lipids, they also lead to modifications of proteins and DNA, and thus ultimately inflicting sperm death. The addition of melatonin to semen extender increases sperm survival rate by preserving membrane integrity and lowering lipid peroxidation of the cryopreserved spermatozoa in a dose‐dependent manner (El‐Raey et al., 2015).

## 5 CONCLUSION

The results obtained from this study suggest that the inclusion of melatonin to the semen freezing media as an antioxidant improved the quality of the cryopreserved bull spermatozoa in terms of mobility and velocity parameters and maintained the fine structure integrity of plasma and acrosome membrane at 0.1 mM melatonin by reducing the cryodamage to spermatozoa. However, in future, cryopreservation and fertilizing potentials studies are needed to confirm the present findings.

## CONFLICT OF INTEREST

None.

## ETHICAL STATEMENT

The authors confirm that the ethical policies of the journal, as noted on the journal's author guidelines page, have been adhered to and the appropriate ethical review committee approval has been received. The Institutional Ethics Committee of Bombay Veterinary College guidelines were followed.
